# Mouse Phenome Database: towards a more FAIR-compliant and TRUST-worthy data repository and tool suite for phenotypes and genotypes

**DOI:** 10.1093/nar/gkac1007

**Published:** 2022-11-04

**Authors:** Molly A Bogue, Robyn L Ball, Vivek M Philip, David O Walton, Matthew H Dunn, Georgi Kolishovski, Anna Lamoureux, Matthew Gerring, Hongping Liang, Jake Emerson, Tim Stearns, Hao He, Gaurab Mukherjee, John Bluis, Sejal Desai, Beth Sundberg, Beena Kadakkuzha, Govindarajan Kunde-Ramamoorthy, Elissa J Chesler

**Affiliations:** The Jackson Laboratory, Bar Harbor Maine, 04609, USA; The Jackson Laboratory, Bar Harbor Maine, 04609, USA; The Jackson Laboratory, Bar Harbor Maine, 04609, USA; The Jackson Laboratory, Bar Harbor Maine, 04609, USA; The Jackson Laboratory, Bar Harbor Maine, 04609, USA; The Jackson Laboratory, Bar Harbor Maine, 04609, USA; The Jackson Laboratory, Bar Harbor Maine, 04609, USA; The Jackson Laboratory, Bar Harbor Maine, 04609, USA; The Jackson Laboratory, Bar Harbor Maine, 04609, USA; The Jackson Laboratory, Bar Harbor Maine, 04609, USA; The Jackson Laboratory, Bar Harbor Maine, 04609, USA; The Jackson Laboratory, Bar Harbor Maine, 04609, USA; The Jackson Laboratory, Bar Harbor Maine, 04609, USA; The Jackson Laboratory, Bar Harbor Maine, 04609, USA; The Jackson Laboratory, Bar Harbor Maine, 04609, USA; The Jackson Laboratory, Bar Harbor Maine, 04609, USA; The Jackson Laboratory, Bar Harbor Maine, 04609, USA; The Jackson Laboratory, Bar Harbor Maine, 04609, USA; The Jackson Laboratory, Bar Harbor Maine, 04609, USA

## Abstract

The Mouse Phenome Database (MPD; https://phenome.jax.org; RRID:SCR_003212), supported by the US National Institutes of Health, is a Biomedical Data Repository listed in the Trans-NIH Biomedical Informatics Coordinating Committee registry. As an increasingly FAIR-compliant and TRUST-worthy data repository, MPD accepts phenotype and genotype data from mouse experiments and curates, organizes, integrates, archives, and distributes those data using community standards. Data are accompanied by rich metadata, including widely used ontologies and detailed protocols. Data are from all over the world and represent genetic, behavioral, morphological, and physiological disease-related characteristics in mice at baseline or those exposed to drugs or other treatments. MPD houses data from over 6000 strains and populations, representing many reproducible strain types and heterogenous populations such as the Diversity Outbred where each mouse is unique but can be genotyped throughout the genome. A suite of analysis tools is available to aggregate, visualize, and analyze these data within and across studies and populations in an increasingly traceable and reproducible manner. We have refined existing resources and developed new tools to continue to provide users with access to consistent, high-quality data that has translational relevance in a modernized infrastructure that enables interaction with a suite of bioinformatics analytic and data services.

## INTRODUCTION

The Mouse Phenome Database (MPD) is an NIH-recognized Biomedical Data Repository focused on primary mouse phenotype and genotype data, giving researchers a stable place to deposit data from individual mice and strains and make it public. This is consistent with NIH data sharing guidelines. MPD has been publicly available since 2001 and has been continuously developed at The Jackson Laboratory (JAX), an independent non-profit research institute that has disseminated mouse genetic data and resources to the biomedical community since its founding. Data are contributed to MPD from researchers around the world—supported by all institutes of the NIH and over 130 research foundations and agencies—and include baseline and treatment data such as drug studies, infectious disease challenges, diet-effect studies, toxicology studies, and studies testing other environmental insults. Data are expertly curated and annotated using Vertebrate Trait Ontology (1), Mammalian Phenotype Ontology (2), Mouse Adult Anatomy Ontology (3), and other controlled vocabularies. Through collaboration with other initiatives, we are working to map these vocabularies and annotations to human disease and human phenotype ontologies. These ontology annotations and other collected metadata allow users to aggregate and analyze data using our modular suite of analysis tools. Datasets are accompanied by detailed protocols so that users can fully understand the data and re-use it confidently or apply the protocols in their own laboratories and compare results to prior work.

MPD houses phenotype and/or genotype data for over 6000 strains and populations. Strain types include inbred, recombinant inbred (including Collaborative Cross strains (4)), F1 hybrid, chromosome substitution, transgenics, and targeted mutants. Populations include offspring from F2 crosses, backcrosses and other experimental crosses, Diversity Outbred mice (5,6), and other heterogeneous stocks such as UM-HET3 mice (7). There are thousands of phenotypic measures alongside a recently developed genotype resource (GenomeMUSter, see below) currently containing observed and imputed genomes for 580+ strains at 83+ million genomic locations, which provides the foundation for genetic meta-analysis across populations and studies (see below).

Human disease areas and conditions that benefit from MPD include addiction, aging, bone and connective tissue disorders, cancer, cardiovascular disease, endocrine/exocrine system disorders, immune function disorders, liver disease, neuromuscular disease, neurodegenerative disease, renal/kidney disease, reproductive conditions, respiratory illness, and others. MPD can be used for many research applications; some use cases are listed in Table 1.

**Table 1.** Common use cases for MPD

Providing validated protocols and relevant data collected under those protocols
Aggregating data based on metadata annotations, e.g. ontology terms, methods used, etc.
Accessing baseline data
Comparing baseline and treatment data
Providing statistical tools that enable:
Choosing optimal strains for different research applications Identifying sensitized strain backgrounds for genetic engineering, e.g. CRISPR Modeling human disease based on multi-dimensional phenotypic profiles Elucidating shared genetics for correlated traits Discovering genotype–phenotype relationships Formulating hypotheses and testing in silico Studying sex differences and sex-by-genotype interactions Assessing replicability across experimental conditions and protocols
Querying one of the largest known public databases of mouse genotype data.

Here, we report new features and advances since our last *NAR* update in 2020 (8). These developments were initiated to make the resource align better to FAIR (9) and TRUST (10) standards, increasing users’ confidence in data re-use and making the data submission process more efficient and standardized.

## NEW FEATURES AND IMPROVEMENTS

### Study intake platform

**Figure 1.**
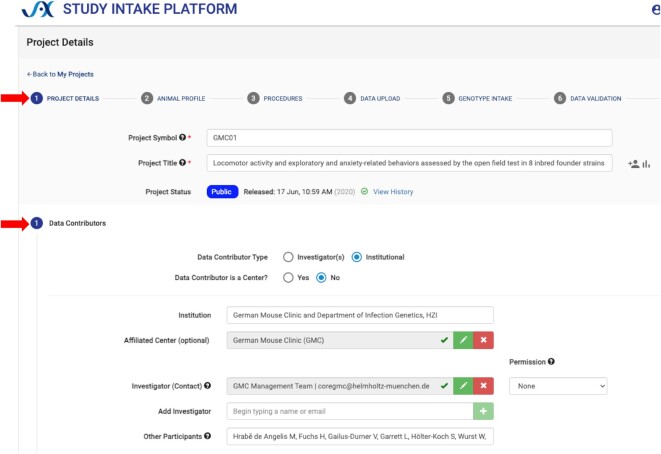
Study Intake Platform screenshot. SIP is set up to be a step-by-step form with tabs (upper red arrow) for Project Details, Animal Profile, Procedures, Data Upload, Genotype Intake and Data Validation. The screenshot shows some of the fields which are available for Project Details (lower red arrow), including information about the contributor (German Mouse Clinic) and participants. The Data Upload tab provides the ability to define data types and study types, and it provides fields for annotating with ontology terms and methods used for each measure uploaded.

The new Study Intake Platform (SIP; https://studyintake.jax.org) was developed for data contributors to self-archive per-animal phenotype data, metadata, and protocol information (registration is required for data contributors to upload data and enter metadata). See screenshot in Figure 1. As domain experts, they can readily annotate their own data with standard ontologies and define data types, experimental designs, and distributional characteristics in a data dictionary, which is used to automate the selection of analysis pipelines applied to that data, e.g. repeated measures, same cohort. The system enables validation of controlled vocabularies and proper nomenclature for mouse strains and other attributes. SIP allows the export of study data in Investigation/Study/Assay (ISA) (11) tab-delimited format. SIP has been enhanced to allow additional data types to be collected. There is now a robust component for collecting genotyping array data from the Neogen platform used for Diversity Outbred and other mouse populations and a mechanism to associate primary raw data files with a study for other molecular phenotypes such as expression data. SIP and MPD share a database and overlapping database schemas.

See more about SIP below in ‘Data Submission’.

### FAIR-compliance and TRUST-worthiness

As a Biomedical Data Repository listed in the Trans-NIH Biomedical Informatics Coordinating Committee registry (https://www.nlm.nih.gov/NIHbmic/domain_specific_repositories.html), MPD accepts submission of relevant data to store, organize, validate, archive, preserve, and distribute the core data to multiple integrative knowledgebases, analysis services and end-users in an increasingly FAIR-compliant (Findable, Accessible, Interoperable and Reusable) (9) and TRUST-worthy (Transparency, Responsibility, User Focus, Sustainability and Technology) (10) manner. By developing the system toward these standards, we have improved data exposure and utilization globally through integration with modernized informatics infrastructure in the research community.

Most of the legacy MPD data application programming interface (API) endpoints have been ported to the new Study Intake Platform (SIP) API to comply to REST standards and includes Swagger documentation. We have investigated various options for registering relevant MPD and SIP data API endpoints with an API management and registration system. We have exposed these endpoints through the JAX-hosted Azure API Management instance, called the JAX BioConnect API Gateway. Most endpoints from both MPD and SIP have been registered with this gateway, with instances for development, Software Quality Assurance (SQA) and external access.

The JAX BioConnect Study Curation Application, an institutional project broadly supporting FAIR data across JAX, acts as a lingua franca for research metadata via the ISA data model. This tool supports the MPD SIP application by gathering and indexing metadata from many study and assay types, and providing selected resources to SIP for specialized curation, data QC, and data analysis. This architectural approach supports FAIR principles in the following ways:

*Findable:* Data are stored and shared in a simple ISA-JSON format, reducing search noise. Data are findable by tools (human and machine-readable API endpoints). Key experimental variables are defined for each study, e.g. factors. Annotations are connected to external, community-supported ontologies. Migration to the Google Cloud Platform (GCP) of the full MPD, SIP, MPD analysis services, and the static document services has been done (see below). MPD is a part of the Registry of Research Data Repositories (re3data; https://www.re3data.org/) and is in the process of registering with identifiers.org (https://registry.identifiers.org/).

*Accessible:* Using JSON-LD, all data files have a unique URL no matter where they are located. For pre-release data, access is controlled via an authorization module for this purpose. Curated study data and well-defined data objects are now more easily understood than ad hoc inconsistent descriptors.

*Interoperable:* There is a registry of tools with their inputs and outputs, supporting arbitrary connections. Ontology annotations facilitate semantic interoperability.

*Reusable:* Versions of software used to generate data files are part of the metadata. Users have access to provenance (who created and processed the data, using what hardware/software, and what processes were used to derive it). Data are exported in a standard RO-Crate format (https://www.researchobject.org/ro-crate/), using Frictionless file type definitions such that data always travels with metadata.

For TRUST guiding principles we have accomplished the following:

*Transparency:* Data submission guidelines and tool and API documentation are available on the MPD website.

*Responsibility:* API has been refined to expose data to external systems using conventional metadata.

*User Focus:* MPD employs user feedback sessions, stakeholder groups, workshops, and webinars (https://www.youtube.com/watch?v=-2c-LWOMRk).

*Sustainability:* Risk mitigation, business continuity, and disaster recovery are provided. Long-term preservation ensures that data remain discoverable, accessible, and usable.

*Technology:* Repository functions are supported by software, hardware, and technical services. Data management and curation are maintained through relevant and appropriate standards, tools and technologies.

Making MPD more TRUST-compliant and TRUST-worthy supports traceability and reproducibility and enables interoperability with other public resources. In the future, measurement, collection, and reporting of data usage for contributors and Google Analytics for the collection of web analytics data and additional industry standard tools for gaining a deeper understanding of the user experience will be employed.

### Phenotype data

#### New content

We have added phenotype data for over 2000 strains of mice, covering hundreds of phenotypes representing nearly all high-level branches of the Vertebrate Trait Ontology. Strain type has been expanded to include targeted mutant strains. Our new tools allow convergence of evidence across heterogeneous datasets (see GWAS Meta-analysis below).

#### Tool to compare metadata across user-selected measures

We have developed a new feature that allows users to quickly compare metadata that has been annotated to a set of user-selected measures. Project information, animal documentation, and experimental details for each measure can be viewed side-by-side in a tabular format for quick comparison. This feature is especially useful when using the GxL Replicability Analysis tool (showcased in the previous NAR update (8)) so that users can optimally choose their measures based on procedural information or diet formulation, for example.

### Genotype data

**Figure 2.**
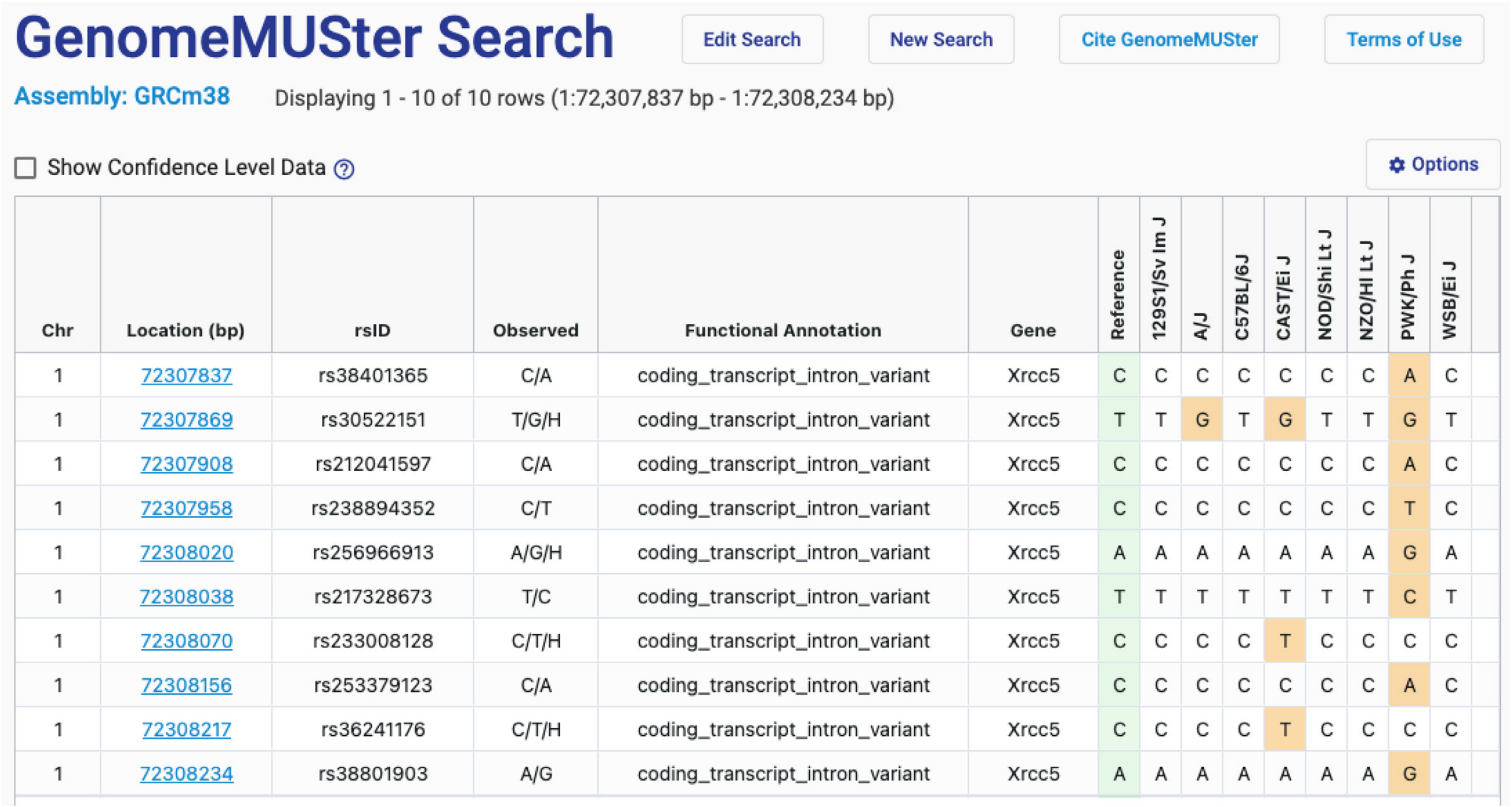
GenomeMUSter search results page. The results table includes chromosome, location (bp), rsID, observed alleles, functional annotation, gene and known and imputed SNP calls for, in this case, Collaborative Cross founder strains. The reference strain is B6Eve (12). Note the option to ‘Show confidence level data’ which provides a heat map. Users can select cut-offs using a handy slider (not shown), and the table will automatically update.

GenomeMUSter (https://mpd.jax.org/genotypes), a new data service hosting known and imputed genotypes, contains data for 581 of the most commonly used laboratory strains of mice at 83+ million genome-wide locations. This database was created by merging 13 genotype datasets: those already hosted through MPD, B6Eve (12), Collaborative Cross (CC) (13) and the Sanger genotype resource released in October, 2020 (REL-2004 v7). Data sources are shown in Table 2. SNPs were imputed using the Viterbi method (14) as implemented in HaploQA (https://haploqa.jax.org). Currently the data are on build GRCm38, and in addition, GRCm39 will be introduced in the near future. Users can query on gene symbol, rs#, or genomic location, with the ability to add upstream and downstream flanking regions. Users select strains one-by-one or more quickly by strain panel, or they can opt to see data for all 581 strains. This tool allows users to browse, visualize, filter on genotype confidence level, and download SNP data. A GenomeMUSter results page is shown in Figure 2. Additional functionality will be added such that a user can compare variation across user defined strain groups. Users will also be able to filter data based on functional annotation. Such functionality is still available for our legacy SNP datasets (listed in Table 2) which do not include access to the millions of new imputed SNPs.

**Table 2.** Sources of genotype data merged for GenomeMUSter

Dataset	Locations	Panel	References
B6Eve^a^	58.8 + K, Chr 1–19, X	Inbred (C57BL/6J Eve)	(12)
Broad2	131K, Chr 1–19, X	Inbred (89)	(15)
CC^a^	43.9 + M, Chr 1–19, X, Y, Mt	Collaborative Cross (69)	(13)^b^
CGD-MDA1	470K, Chr 1–19, X, Y, Mt	Inbred (142)[	(16)
CGD-MDA2	470K, Chr 1–19, X, Y, Mt	BXD w/ parents (92)	(16)
CGD-MDA3	470K, Chr 1–19, X, Y, Mt	ILSXISS w/ parents (69)	(16)
CGD-MDA4	470K, Chr 1–19, X, Y, Mt	AXB, BXA, BXH, CXB, AKXL w/ parents (72)	(16)
CGD-MDA5	470K, Chr 1–19, X, Y, Mt	B6.A, B6.PWD (53)	(16)
Perlegen2	8.1 + M, Chr 1–19, X, Y, Mt	Inbred (16)	(17)
Sanger^a^	79.5 + M, Chr 1–19, X, Y	Inbred (53)	(18)^c^
UCLA1	132K, Chr 1–19, X	Hybrid Mouse Diversity Panel (248)	^d^
UNC-GMUGA1	130K, Chr 1–19, X, Y, Mt	Collaborative Cross w/ parents (77)	(19)
UNC-MMUGA2	76K, Chr 1–19, X, Mt	Collaborative Cross w/ parents (77)	(13)

^a^Not an MPD SNP legacy dataset.

^b^ https://csbio.unc.edu/CCstatus/CCGenomes/.

^c^REF-2004 v7, 12 October 12020.

^d^ http://mouse.cs.ucla.edu/mousehapmap/emma.html.

### Phenotype-genotype association

**Figure 3.**
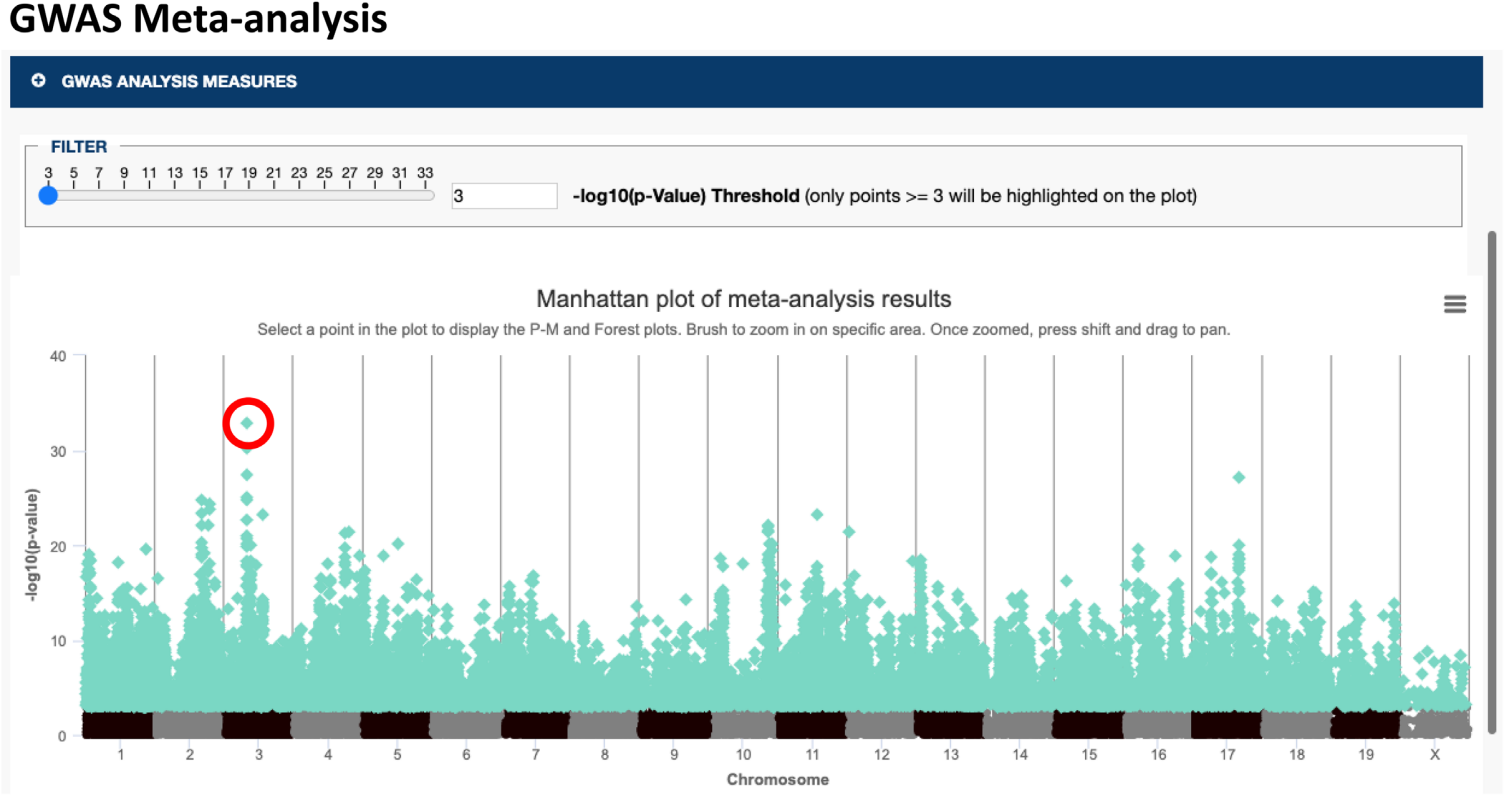
Manhattan plot of GWAS meta-analysis results. Bone mineral density measures (*n* = 10 from seven studies) were chosen for analysis (see accession numbers). Genomic location is displayed along the x-axis and the negative logarithm (base 10) of the association *P*-value for each SNP is shown on the y-axis. Note that data points ≥3 are highlighted and results can be filtered based on –log_10_(*P*-value) (see slider). The circled SNP is further analyzed in Figure 4.

**Figure 4.**
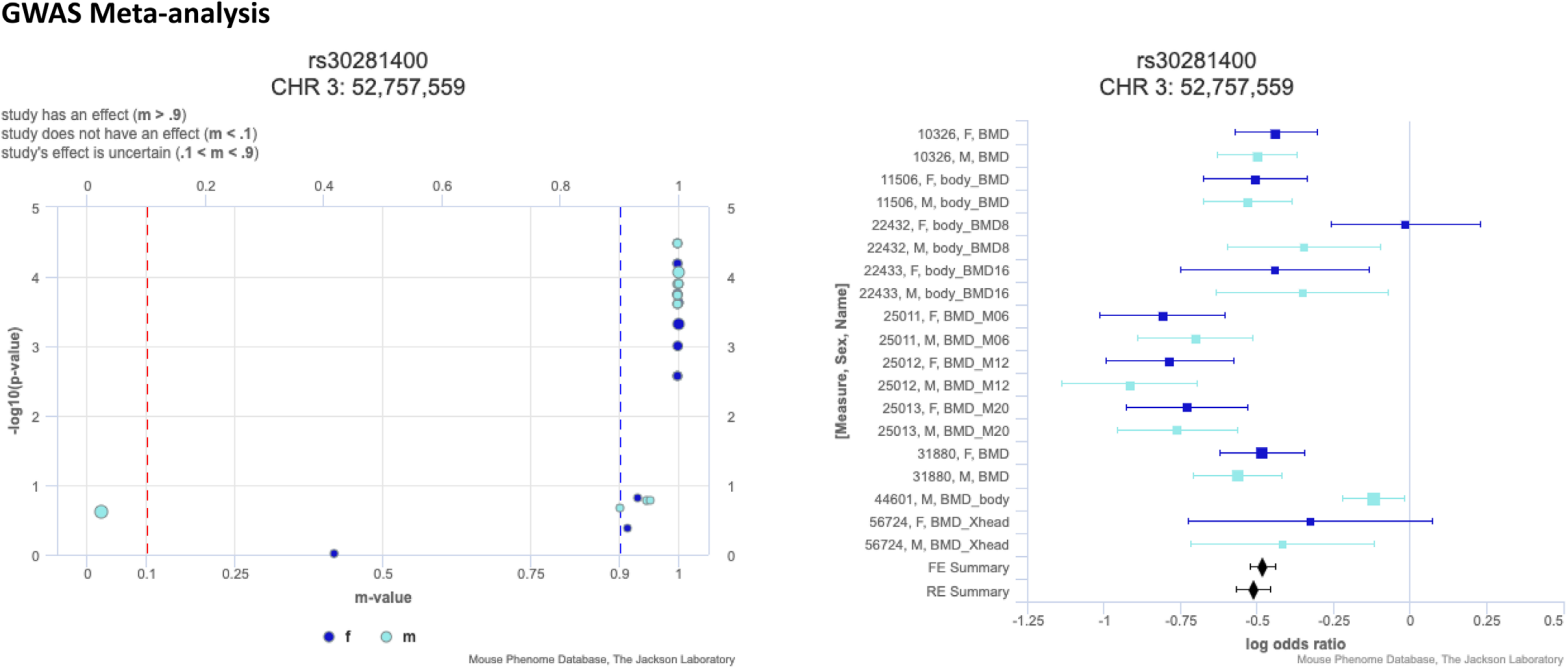
P–M plot (left) and Forest plot (right) for SNP rs30281400, circled in Figure 3. For a selected SNP, the P–M plot displays the individual measures’ *P*-values versus the m-values (posterior probability the effect exists in the measure) (22). The m-value distinguishes measures where the effect exists (*m*-value ≥ 0.9), measures where the effect does not exist (*m*-value ≤ 0.1), and measures where the effect is uncertain (0.1 < *m*-value < 0.9). The Forest plot shows effect size (midline), standard error (bars), and study size (size of box) on a log odds ratio axis for all measures in the analysis. Data are color coded based on sex.

GWAS meta-analysis has become an important tool for genetic association studies to identify variants that affect a trait (or set of traits) of interest by pooling power across studies and populations. Combined studies are often heterogeneous due to differing environmental conditions, populations, and study designs so we implemented variant level visualizations of meta-analysis results to investigate if the effect is broad or specific to certain conditions or methods of measurement. We have implemented the open-source software tool METASOFT (http://genetics.cs.ucla.edu/meta/) (20,21), a meta-analysis software program based on a random-effects model, into our suite of analysis tools. METASOFT output data include mixed effect meta-analysis *P*-values for a set of user-selected measures which can be visualized in a Manhattan plot where genomic location is displayed along the x-axis and the negative logarithm (base 10) of the meta-analysis *P*-value for each SNP is plotted along the y-axis (Figure 3). METASOFT achieves high statistical power while it also corrects the population structure by employing mixed effect models. For a selected SNP, we investigate if the effect is broad or specific to a condition, sex, or other factors using P–M and Forest plots (20–22) (Figure 4).

To use the GWAS Meta-analysis tool, a user must first select measures of interest (minimum of four) and the eligible tools for those measures will be made available in the toolbox. To see a demo of this tool, go to the ‘Tools Demo’ page (https://phenome.jax.org/tools/phenomenu?demo=1) and follow the directions for selecting the appropriate measures for this tool.

## FUTURE DIRECTIONS

We will continue our efforts to become even more FAIR-compliant and TRUST-worthy, including the adaptation of the Phenopackets standard (23) for MPD measures to link them to detailed phenotypic descriptions and thereby streamline exchange and systemic use of mouse phenotype data. Extension of our APIs allow integration with GenomeMUSter and will facilitate interoperability with other tools in external resources. We will be adding more functionality and diversity for the GWAS Meta-analysis tool, including additional visualizations. We will also take advantage of the Mouse-Human Ontology Mapping Initiative (https://github.com/mapping-commons/mh_mapping_initiative) which aims to integrate human and mouse phenotype data via dominant controlled vocabularies (Human Phenotype Ontology and Mammalian Phenotype ontology). When this has been done, we will be able to annotate and aggregate measures with disease terms. We already utilize SciCrunch Research Resource identification numbers (RRIDs) (24) for experimental resources. We will add RRIDs for strains and panels which will enable data sharing with other resources also using RRIDs. Finally, we will develop a tool to send variant sets generated by the GenomeMUSter tool and GWAS Meta-analysis tool to GeneWeaver (25) and its variant graph database to ultimately identify mouse genes functionally related to the variants, including human genes and variants of orthologous effect, which may influence related trait variation (26).

## ACCESSING AND SUBMITTING DATA

### Bulk and programmatic access

A set of public API endpoints is available for programmatic access to specific phenotype data (individual animal data or strain means), metadata, and analytics results (all returned in JSON or csv format). For more information, see https://phenome.jax.org/about/api. We are making our API endpoints available through an API Gateway, with full documentation of endpoints and parameters. The JAX BioConnect team is building infrastructure that allows the generation and download of all studies in RO-Crate format. SNP data from GenomeMUSter can also be downloaded, browsed, and visualized. As in the past, bulk data downloads are available at https://phenome.jax.org/downloads.

### Data submission

The new Study Intake Platform is ready for data contributors wanting to submit strain survey data to be housed in MPD. Simply go to the SIP homepage to get started (https://studyintake.jax.org). Registration is required to be a data contributor. This is necessary to provide contributors viewing/editing privileges for their data/study which is kept private until the investigator is ready to release it to the public. On the SIP homepage, go to the ‘?’ icon in the left menu for detailed guidelines on how to structure data sets and load data and metadata. There is also FAQ. A quick-start guide will be added in the near future. Users can contact phenome@jax.org for support in curation or use of the applications. Alternatively, data and metadata can be expertly curated by a professional biocurator accessed via phenome@jax.org.

## IMPLEMENTATION AND PUBLIC ACCESS

As of this writing, the MPD team has migrated its ecosystem to Google Cloud Platform (GCP) and now no longer relies on on-premises hardware resources. The following application parts are deployed in GCP using a Kubernetes cluster: the MPD web application, the Study Intake Platform, and the MPD analysis service (does not have direct external visibility). The MPD web application is a legacy Python Flask application that renders pages using Jinja2 templates in HTML and JavaScript. SIP is a Python Flask RESTplus application, providing user access through an Angular web application and program access via REST endpoints. The database for MPD and SIP is a PostgreSQL database that is hosted in the cloud using CloudSQL. The MPD Analysis Server is a Python RESTplus web service application with analytics implemented in both Python leveraging Pandas and via rpy2 to call an R-based mpdanalysis package implemented by data analysts and statisticians on the MPD team. The GWAS Meta-analysis Service is a cloud-based server which runs analyses using Temporal.io pipelines. The meta-analysis library and the service which controls it are written in Java. The data store and API to the GenomeMUSter application have been implemented in GCP using BigQuery.

We have been re-architecting all legacy aspects of the application and implement it using an Angular client, with PrimeNG components and styling, that uses REST API services from a collection of resources including SIP-API, the BioConnect Study Curation service, the MPD Analysis Server, the MetaAnalysis Server, GenomeMUSter, and a newly implemented REST-based FastAPI Python server for MPD-specific data and aggregations of data for tools. The user interface to GenomeMUSter has already been implemented as the prototype for the new MPD, using Angular.

All our GCP Kubernetes applications are deployed in two clusters. The first is our development and testing cluster which has both a development and SQA instance. The second is our production cluster which has a staging and production instance. MPD developers can deploy updates to the ‘dev’ and ‘sqa’ environments. All code goes through a Pull Request based code review process prior to being deployed to SQA, at which point our SQA team tests all software before deployment to the staging environment for final review and testing, followed by release to our production environment. In addition, there have been improved security updates (content-security-policy) and automation of data loading for ontologies and LIMS data.

## CITING MPD

For a general citation of the MPD resource, this NAR article should be cited and use RRID:SCR_003212. The following citation format is suggested when referring to MPD datasets: Investigator(s) name(s) [last name, first initial, middle initial]. Title of project. MPD:project symbol [such as Jones1]. Mouse Phenome Database web resource (RRID:SCR_003212), The Jackson Laboratory, Bar Harbor, Maine USA. https://phenome.jax.org [Cited (date)].

## DATA AVAILABILITY

RO-Crate format (https://www.researchobject.org/ro-crate/); METASOFT (http://genetics.cs.ucla.edu/meta/); Mouse-Human Ontology Mapping Initiative (https://github.com/mapping-commons/mh_mapping_initiative); HaploQA (https://haploqa.jax.org); Collaborative Cross Genomes (https://csbio.unc.edu/CCstatus/CCGenomes/); MPD SNP legacy datasets (https://phenome.jax.org/about/snp_retrievals_help).

The SciCrunch Research Resource (24) identification number for MPD is RRID:SCR_003212. Accession numbers for MPD data used for the GWAS Meta-analysis tool (Figures 2 and 3) are as follows: MPD:10326, MPD:11506, MPD:22432, MPD:224333, MPD:25011, MPD:25012, MPD:25013, MPD:31880, MPD:44601, MPD:56724.
